# Investigation of microRNAs in mouse macrophage responses to lipopolysaccharide-stimulation by combining gene expression with microRNA-target information

**DOI:** 10.1186/1471-2164-16-S12-S13

**Published:** 2015-12-09

**Authors:** Chia-Chun Chiu, Wei-Sheng Wu

**Affiliations:** 1 Department of Electrical Engineering, National Cheng Kung University, No.1 University Road, Room 92879, 701 Tainan, Taiwan

**Keywords:** microRNA, macrophage, inflammation, immune system, toll-like receptor, lipopolysaccharide

## Abstract

**Background:**

Toll-like receptors, which stimulated by pathogen-associated molecular patterns such as lipopolysaccharides (LPS), induces the releasing of many kinds of proinflammatory cytokines to activate subsequent immune responses. Plenty of studies have also indicated the importance of TLR-signalling on the avoidance of excessive inflammation, tissue repairing and the return to homeostasis after infection and tissue injury. The significance of TLR-signalling attracts many attentions on the regulatory mechanisms since several years ago. However, as newly discovered regulators, how and how many different microRNAs (miRNAs) regulate TLR-signalling pathway are still unclear.

**Results:**

By integrating several microarray datasets and miRNA-target information datasets, we identified 431 miRNAs and 498 differentially expressed target genes in bone marrow-derived macrophages (BMDMs) with LPS-stimulation. Cooperative miRNA network were constructed by calculating targets overlap scores, and a sub-network finding algorithm was used to identify cooperative miRNA modules. Finally, 17 and 8 modules are identified in the cooperative miRNA networks composed of miRNAs up-regulate and down-regulate genes, respectively.

**Conclusions:**

We used gene expression data of mouse macrophage stimulated by LPS and miRNA-target information to infer the regulatory mechanism of miRNAs on LPS-induced signalling pathway. Also, our results suggest that miRNAs can be important regulators of LPS-induced innate immune response in BMDMs.

## Background

The immune system is composed of innate and adaptive immune systems, in which the machinery behind innate immune system is very ingenious due to the conserved ability to recognize microbial components during evolution process. Toll-like receptors (TLRs) are a kind of specialized sensor responsible for recognizing molecular patterns of pathogens and bridging innate and adaptive immunity by activation of antigen-presenting cells such as dendritic cells and macrophages. Upon exposure of molecular patterns of pathogens, TLRs initiate a cascade of intracellular signalling transduction that leads to secretion of inflammatory mediators, including cytokines and chemokines, inducing expression changes of a broad spectrum of genes that regulate defense function against pathogens.

TLR4, the best characterized member of TLR family, can detect lipopolysaccharide (LPS) from Gram-negative bacteria. Once TLR4 is stimulated by LPS, two independent signalling pathways (TRIF-dependent and MyD88-dependent) will be initiated, and many import transcription factors (TNF-α, IFN-β, IL-1β, etc.) will be activated as well. Although several mechanisms which are responsible for the regulation of TLR4-induced signalling pathways have been already well-studied, including physical interactions, changes of conformation, phosphorylation, ubiquitylation, and etc [1-3], how an anti-inflammatory response is induced and the processes by which inflammation remain unclear [4]. Among these regulators, miRNAs have drew enough notice as a newly identified regulators in last several years.

MicroRNAs (miRNAs), a class of small non-coding RNAs, has emerged as important regulators in the development of immune and inflammatory responses [4-8]. The importance of miRNAs in the modulation of normal and pathological immune function has been shown in multiple studies in which deregulation of miRNAs was demonstrated to accompany diseases associated with excessive or uncontrolled inflammation. As a critical part of regulatory networks in innate immunity, the dysregulation of some specific miRNAs would modulate the regulation of inflammatory in stimulated immune cells. According to the literature [9], many miRNAs are reported to be dys-regulated in activated immune cells, such as miR-146a.

Recently, many studies have attempted to develop methods to understand miRNA co-operativity. Most of these studies however did not actually considered coexpression profiles of mRNAs and miRNAs. Considering that most miRNAs exert their functions through interactions with other miRNAs, an understanding of a miRNA network context using both co-expression pattern and the sequence complementarity between miRNAs and mRNAs is essential to discover the cooperative regulation of miRNAs.

In this paper, we used two kinds of data sets to infer the regulatory mechanism of miRNAs on LPS-induced signalling pathway. mRNA expression data and miRNA-target information data are used to identify reliable regulatory relationship between miRNAs and mRNA, and then cooperative miRNAs can be identified using hypergeometric test. Besides, all differentially expressed target genes were used to perform coherent functional analysis, the possible function of miRNAs which actually regulate gene expression could be hence inferred. Using these cooperative miRNAs and their common target genes, a cooperative miRNA network could be constructed and the cooperative miRNA modules could be also identified.

## Materials and methods

### Overview

The work flow for identification of co-regulating miRNAs in mouse macrophages with stimulation of LPS. Microarray expression data and miRNA-target information data are used to identify reliable regulatory relationship between miRNAs and mRNA, and then cooperative microRNAs can be identified using hypergeometric test. Besides, all differentially expressed target genes were used to perform coherent functional analysis, the possible function of miRNAs which actually regulate gene expression could be hence inferred. Using these cooperative miRNAs and their common target genes, a cooperative miRNA network could be constructed and the cooperative miRNA modules could be also identified.

### Datasets

In this study, five expression profile datasets and four miRNA-target information datasets are used. Expression datasets, which were downloaded from NCBI GEO database [10], are from five experiments generated individually by Barish *et al *[11], El Chartouni *et al *[12], Ghigo *et al *[13], Mages *et al *[14] and Millien *et al *[15], which were downloaded from NCBI GEO database [10]. Barish *et al *used Illumina MouseWG-6 v2.0 expression beadchip to compare the changes of gene expression between wildtype and Bcl6 KO bone marrow-derived macrophages of mouse in the absence or presence of LPS (GSE24792). El Chartouni *et al *used Agilent-014868 Whole Mouse Genome Microarray to study TLR4 induced transcriptional responses in interleukin 4 primed mouse bone marrow-derived macrophages (GSE21895). Ghigo *et al *used Agilent-014868 Whole Mouse Genome Microarray to compare the difference of gene expression of mouse bone marrow-derived macrophage response to T. whipplei and LPS (GSE20210). Magges *et al *used Affymetrix Mouse Genome 430 2.0 Array to study the development of a state of refractoriness to a second stimulation in mouse bone marrow-derived macrophages treated with LPS (GSE8621). Millien *et al *used Illumina MouseWG-6 v2.0 expression beadchip to study the effects of mouse bone marrow derived macrophages treated with PAO, IFN-gamma, or LPS (GSE48609). On the other hand, miRNA-target gene interaction datasets were downloaded from Mouse Genome Informatics (MGI) database [16]. These datasets mainly come from four different data sources, in which miRTarBase [17] data are validated, but microt-cds [18], miRDB [19] and Pictar [20] data are all predicted. The list of mouse genetic markers which also downloaded from MGI database was used as the list of all genes and miRNA in mouse.

### Expression data preprocessing and differentially expressed genes identification

In each expression dataset, only wild-type samples in the absence or presence of LPS were used in subsequent data analyses. The value distribution service of GEO2R in NCBI GEO database was used to determined if this data are normalized and cross-comparable, and the results revealed that only the datasets from Barish *et al *study and from Millien *et al *study are not needed to be normalized. Other three datasets were quantile-normalized using R package "affyPLM" [21]. For every expression datasets, differential expression analyses were performed using R package "limma" [22]. Benjamini-Hochberg method was used to control the false discovery rate (FDR), and genes with adjusted p-value less than 0.05 were identified as differentially expressed (DE) genes.

### Preprocessing of miRNA-target gene interaction data

Due to a large part of miRNA-target gene interaction data consists of predicted data, there are many false positives in these datasets. Therefore, only the data from miRTarBase and the data appear at least in two of three predicted data sources were retained, and then we obtained a list with 822697 miRNA-target gene interaction data. We obtained 712 miRNAs from this list, and then remove the miRNAs which could be found in the list of mouse genetic markers. Finally, 703 miRNAs were retained.

### Gene set enrichment analysis

We carried out the gene set enrichment analysis to identify the miRNAs which actually participate in the regulation of target genes. For each miRNA, a 2 × 2 contingency table was built (whether the genes are targets of this miRNA or not versus whether the genes are differentially expressed) to access the enrichment level using Fisher's exact test. After all miRNAs were tested, Benjamini-Hochberg procedure for multiple test correction was used to correct p-values. This miRNA would be considered to have a significant number of differentially expressed target genes if the adjusted p-value of Fisher's exact test is less than 0.05, and this miRNA could be thus considered a reliable regulator in mouse macrophages under LPS-stimulated condition.

### Coherent functional analysis

Cytoscape v3.2.1 (http://cytoscape.org/) with ClueGO v2.1.6 plug-in [23] was used to perform GO and KEGG pathway analyses. ClueGO let us to obtain the distribution of the list of differentially expressed target genes across the GO terms and pathways. We used right-sided hypergeometric test to calculate enrichment level and used Benjamini-Hochberg adjustment to correct p-values in multiple test. A GO term or pathway would be considered to be enriched in differentially expressed target genes if its adjusted p-value is less than 0.05. We analyzed up-regulated target genes and down-regulated target genes using these settings in GO and KEGG pathway analyses, respectively.

### Construct cooperative miRNA network

Cooperative miRNA network (CMN) is defined as a network composed of cooperative miRNAs sharing a significant number of differentially expressed target genes, which could be used to grouping these cooperative miRNAs. The assumption behind CMN is based on the concepts of co-expressed and co-regulation, which means that if two regulators are co-operative, the two regulators should share a significant number of common target genes with similar expression profiles. In a CMN, each node represents a specific miRNA and each edge represents a specific co-operativity between a pair of miRNAs. We performed significance analysis using hypergeometric test as follows:

(1) $${\text{P}}_{\text{overlap}}=\overset{\text{min}\left(N_{i},\;N_{j}\right)}{\underset{k=m}{\sum }}\frac{C_{k}^{N_{i}}C_{N_{j}-k}^{N-N_{i}}}{C_{N_{j}}^{N}}$$

, where *N_i _*and *N_j _*are the number of differentially expressed target genes of miRNA *i *and of miRNA *j*, respectively. *P_overlap _*is the p-value on this test, *N *is the total number of DE genes and *m *is the number of common differentially expressed target genes between miRNA *i *and miRNA *j*. Subsequently, Benjamini-Hochberg method was used to adjust p-values and the miRNA pair with adjusted p-value less than 0.01 was considered as sharing a significant number of common differentially expressed target genes. After these significance analyses, a list of cooperative miR-NAs would be obtained. Next step, a target overlap score was used to represent the weight of each edge in the CMN by calculating the Jaccard similarity coefficient. The *P_overlap _*or −*logP_overlap _*value were not used as overlap scores due to the large difference between these p-values. The target overlap score according to the definition in Na and Kim's paper [24] is defined as follows:

(2) $${\text{S}}_{\text{overlap}}=\left\{\begin{array}{cc} \frac{\left|\text{Targets}\left(i\right)\cap \text{Targets}\left(j\right)\right|}{\left|\text{Targets}\left(i\right)\cup \text{Targets}\left(j\right)\right|} & ,i\neq j\\ 1 & ,i=j \end{array}\right)$$

, where *Targets*(*i*) and *Targets*(*j*) represent the set of target genes of miRNA *i *and miRNA *j*, respectively. As a result, the list of cooperative miRNAs and the target overlap scores were used to construct CMN.

### Extraction of cooperative miRNA modules (CMM)

In this paper, a sub-network detection algorithm, Molecular COmplex DEtection algorithm (MCODE) [25], was applied to find possible coherent modules in the global cooperative miRNA network. The MCODE, which is a clustering algorithm based on graph theorem, is specifically designed to find complexes by identifying densely connected subgraphs in networks. MCODE algorithm is composed of three stages: vertex weighting, complex prediction and an optional post-processing step. The core clustering coefficient is used as the weights of nodes. Once the weights are computed, the algorithm traverses the weighted graph using greedy algorithm to isolate densely connected regions. The post-processing step filters or adds nodes based on connectivity criteria. MCODE has a parameter that specifies the size of clusters returned. All parameters in MCODE are applied with default values. Sub-networks are filtered if they do not contain at least a 2-core (graph of minimum degree 2). This approach allows us to assign one miRNA to multiple clusters, considering a biological principle that miRNAs can have multiple functions.

## Results

### Differentially expressed mRNA

**Figure 1:**
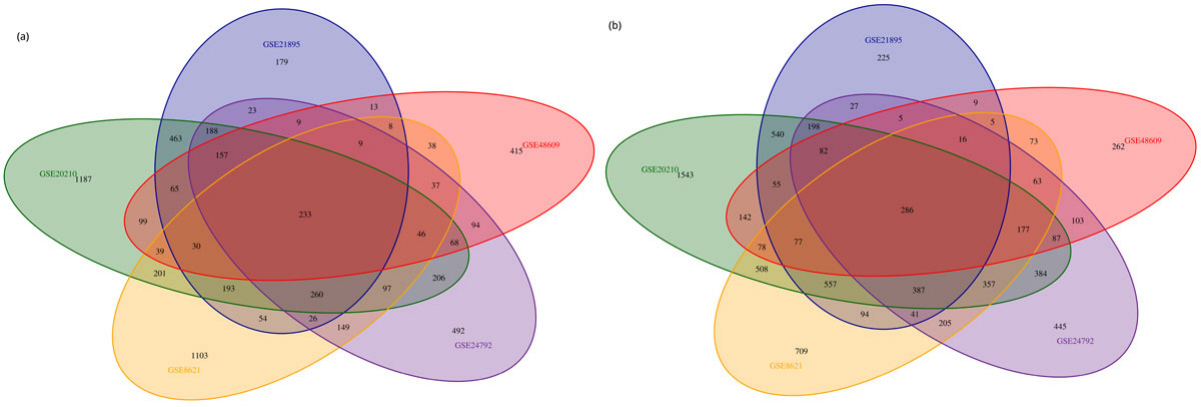
**The intersection of up-regulated and down-regulated DE genes among five expression datasets**.

After differential expression analyses, we obtained 8990 DE genes from GSE20210 dataset, in which 3532 genes are up-regulated and 5458 genes are down-regulated. Similarly, there are 4514 DE genes (including 1910 up-regulated genes and 2604 down-regulated genes) identified in GSE21895 dataset, 4597 DE genes (including 2094 up-regulated genes and 2863 down-regulated genes) identified in GSE24792 data set, 2880 DE genes (including 1360 up-regulated genes and 1520 down-regulated genes) identified in GSE48609 dataset, and 6516 DE genes (including 2523 up-regulated genes and 3633 down-regulated genes) identified in GSE8621 dataset. In order to avoid the problems caused by bias between different platforms, we only used the intersection of the results from these five expression datasets, which cause that 233 up-regulated genes and 286 down-regulated genes left (see Figure 1). Besides, any differentially expressed probe from any platform which could not be mapped to any Entrez gene ID or any gene does not appear in the list of miRNA-target gene interaction were removed. Finally, 228 up-regulated genes and 270 down-regulated genes were retained for subsequent analyses.

### GO and KEGG enrichment of differentially expressed genes

**Figure 2:**
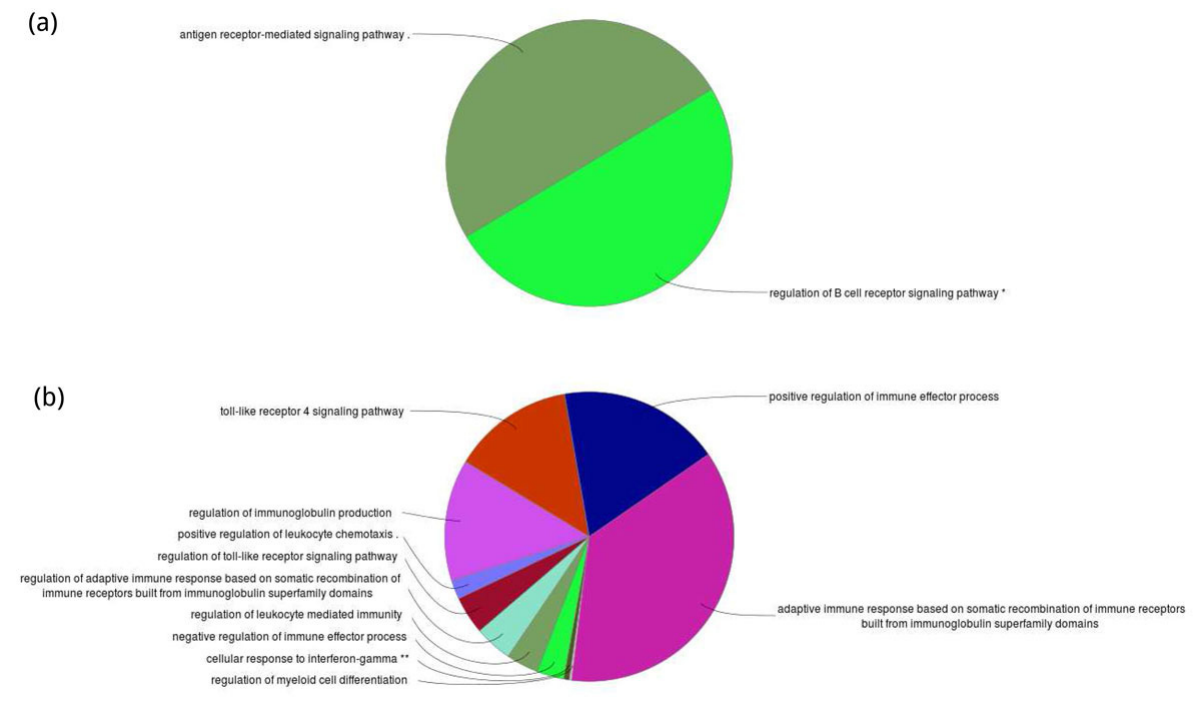
**The results of GO enrichment analyses on DE genes**. (a) The number of enriched GO terms in down-regulated genes is 6, and these GO terms can be classified into two classes. (b) The number of enriched GO terns in up-regulated genes is 152, and these terms can be classified into 11 class.

**Figure 3:**
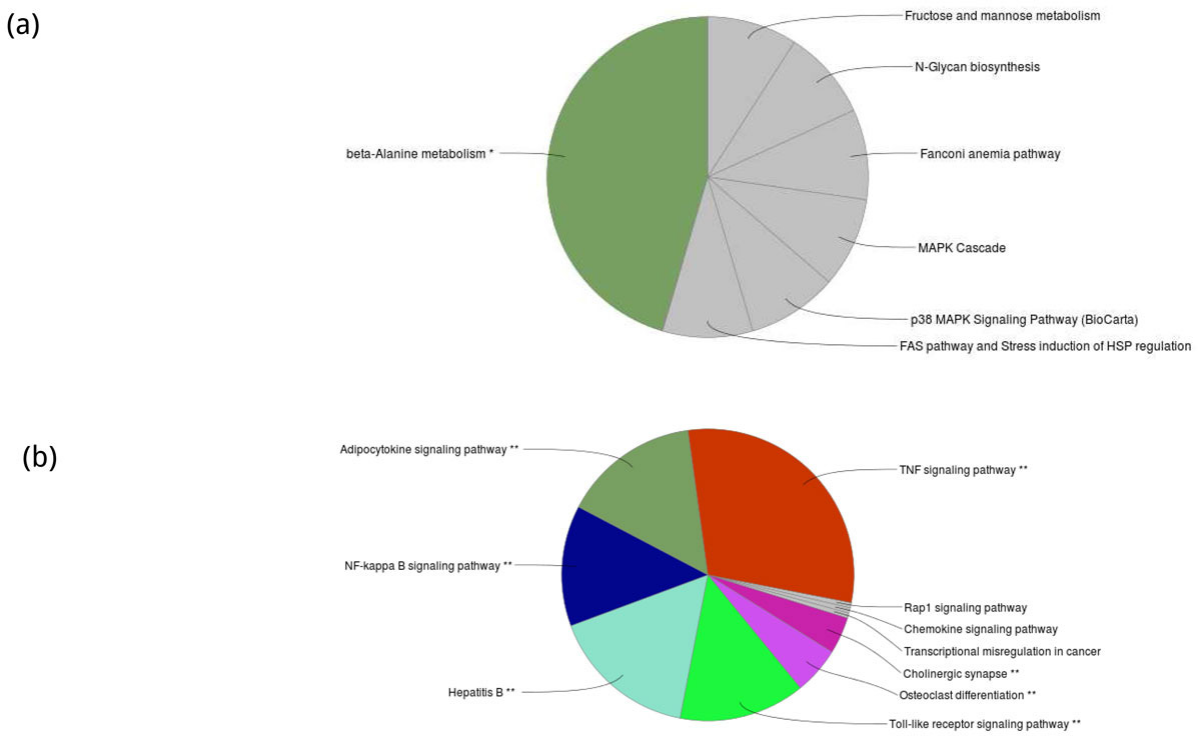
**The results of KEGG enrichment analyses on DE genes**. (a) The number of enriched KEGG pathways in down-regulated genes is 11, and (b) the number of enriched KEGG pathways in up-regulated genes is 78.

In this study, we performed KEGG-enrichment analysis and GO-enrichment on differentially expressed genes separately. For down-regulated genes, they enrich in two different pathways: antigen-receptor and B cell receptor signalling pathways (see Figure 2a). Besides, we found that these down-regulated genes enrich in several immune system processes, including adaptive immune response based on somatic recombination of immune receptors built from immunoglobulin super-family domains, positive regulation of immune effector process, toll-like receptor 4 pathways, regulation of immunoglobulin production, regulation of toll-like receptor signalling pathway, negative regulation of immune effector process, regulation of leukocyte mediated immunity and so on (see Figure 2b). On the other hand, seven different functional pathways were found enriched in up-regulated genes, including β-Alanine metabolism, fructose and mannose metabolism, N-glycan biosynthsis, Fanconi anemia pathway, MAPK cascade, p38 MAPK signalling pathway and FAS pathway/Stress induction of HSP regulation (see Figure 3a). After GO-enrichment analysis, 10 immune system process were found enriched in up-regulated genes, including Adipocytokine signaling pathway, NF-κB signalling pathway, Hepatitis B, TNF signalling pathway, Rap1 signalling pathway, Chemokine signalling pathway, transcriptional mis-regulation in cancer, Cholinergic synapse, osteoclast differentiation and toll-like receptor signalling pathway (see Figure 3b).

### Construction and characterization of cooperative miRNA networks

**Figure 4:**
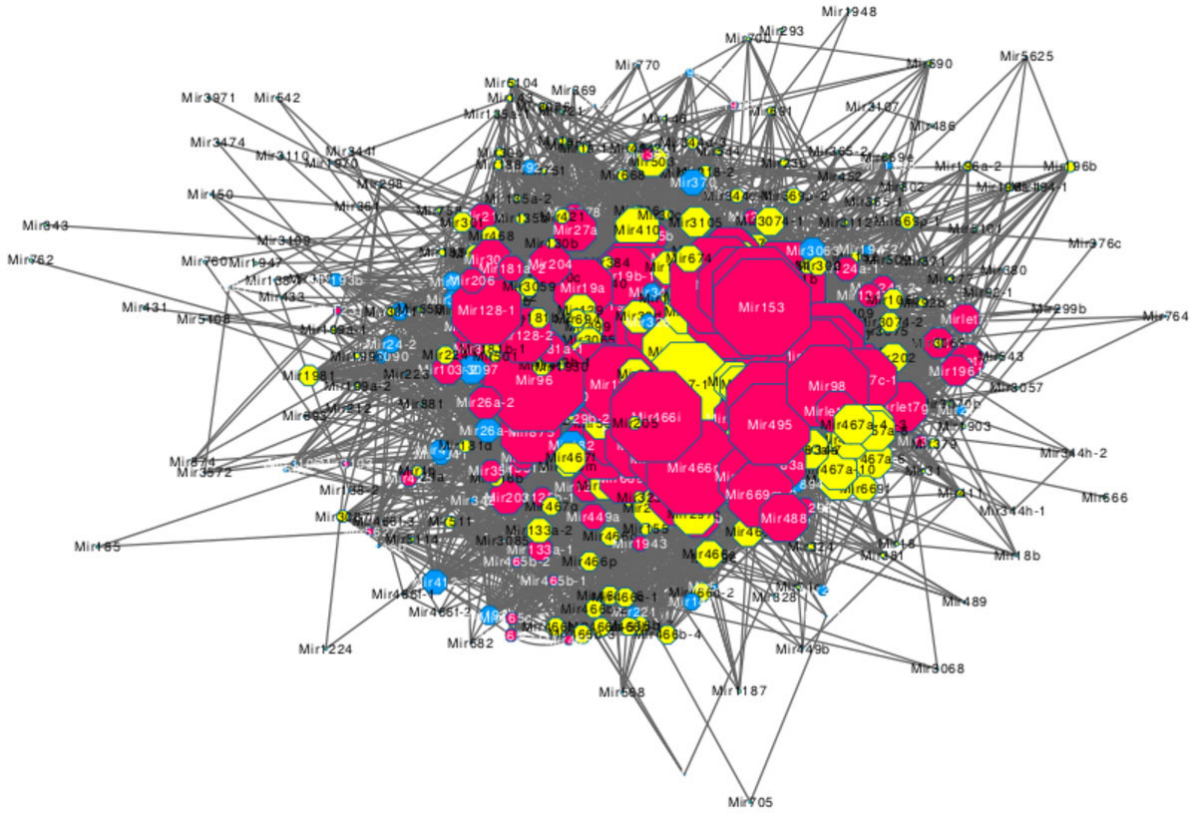
**The superposition of CMNs which composed of microRNAs regulating up-regulated genes and down-regulated genes**.

Figure 4 shows the global CMN created by superimposing CMN composed of miR

NAs up-regulating target genes and CMN composed of miRNAs down-regulating target genes, there exists 417 nodes and 7901 edges. In CMN, each node corresponds to a miRNA that has a significant number of differentially expressed target genes in mouse macrophage with the stimulation of LPS, and each edge represents that the miRNA pair on both side of this edge share a significant number of common differentially expressed target genes. The nodes with yellow colour are the miRNAs which only up-regulate genes, the nodes with red colour are the miRNAs which only down-regulate genes, and the nodes with blue colour are the miRNAs which up-regulate and down-regulate genes, which implies that there may exists a more complex mechanism behind the regulation of innate immunity. Using MCODE on CMN composed of miRNAs up-regulating target genes, 17 cooperative miRNA modules were found (Table 1). The numbers of miRNAs and connections of CMMs range from 3 to 43 and 3 to 696, respectively. While using MCODE on CMN composed of miRNAs down-regulating target genes, 8 cooperative miRNA modules were found (Table 2) and the numbers of miRNAs and connections of CMMs range from 3 to 34 and 3 to 374, respectively. This result shows that most of miRNAs are multiple connected, and most of miRNAs do not own significantly more edges than others, which means that most of miRNAs must act co-operatively.

**Table 1:** Cooperative miRNA modules in which miRNAs up-regulate genes.

Cluster	Score	Nodes	Edges	miRNAs
1	33.143	43	696	Mir106b, Mirlet7b, Mir98, Mir297c, Mir9-1, Mir9-2, Mir200b, Mir467d, Mir9-3, Mir669f, Mir467c, Mir466n, Mir297a-4, Mir669m-2, Mir669m-1, Mir302a, Mir467b, Mirlet7a-1, Mir200a, Mir141, Mir297a-3, Mir669l, Mirlet7c-2, Mirlet7f-1, Mir467a-10, Mir467f, Mir467a-9, Mir669d-2, Mir669d, Mir467e, Mir669b, Mir467a-4, Mir93, Mir467a-3, Mir467a-1, Mir467a-2, Mir297b, Mir302d, Mir17, Mir467a-8, Mir467a-7, Mir467a-6, Mir467a-5

2	14.067	31	211	Mir294, Mir295, Mir302c, Mir466c-2, Mir466c-1, Mir466p, Mir291b, Mir291a, Mir466, Mir466e, Mir467g, Mir466b-4, Mir466m, Mir292, Mir466b-3, Mir466b-7, Mir466b-6, Mir466b-5, Mir466b-8, Mir539, Mir493, Mir466o, Mir106a, Mir20bm Mir302b

3	9.4	21	94	Mir20a, Mir153, Mir202, Mir322, Mirlet7i, Mirlet7c-1, Mir466i, Mirlet7e, Mir7b, Mir22, Mir7-2, Mirlet7f-2, Mir1961, Mirlet7d, Mir488, Mir19b-1, Mir320, Mir669p-1, Mir196b, Mir669p-2, Mirlet7a-2

4	8.952	43	188	Mir503, Mir216a, Mir883a, Mir421, Mir883b, Mir551b, Mir5619, Mir30c-2, Mir3065, Mir3069, Mir410, Mir128-2, Mir5624, Mir103-1, Mir742, Mir344e, Mir344d-2, Mir133a-2, Mir3074-1, Mir181b-2, Mir133a-1, Mir499, Mir743, Mir497, Mir3082, Mir300, Mir344d-1, Mir344d-3, Mir124a-3, Mir30b, Mir124a-1, Mir124a-2, Mir495, Mir448, Mir101a, Mir188, Mir30c-1, Mir27b, Mir382, Mir301b, Mir374c, Mir323, Mir384

5	7.29	32	113	Mir494, Mir429, Mir15b, Mir128-1, Mir5616, Mir1907, Mir107, Mir30a, Mir33, Mir103-2, Mir140, Mir222, Mir181b-1, Mir3074-2, Mir7-1, Mir216b, Mir181d, Mir16-1, Mir19b-2, Mir468, Mir15a, Mir743b, Mir96, Mir181c, Mir1912, Mir214, Mir674, Mir1906-1, Mir582, Mir27a, Mir1906-2, Mir301

6	5.167	25	62	Mir465b-1, Mir152, Mir32, Mir367, Mir465, Mir218-1, Mir465c-2, Mir465c-1, Mir758, Mir129-1, Mir30d, Mir129-2, Mir3066, Mir706, Mir204, Mir669c, Mir19a, Mir186, Mir465b-2, Mir1192, Mir875, Mir92b, Mir16-2, Mir669h, Mir673

7	4	4	6	Mir694, Mir199a-2, Mir199a-1, Mir199b

8	3.333	4	5	Mir212, Mir351, Mir125b-1, Mir125b-2

9	3	13	18	Mir449a, Mir218-2, Mir449b, Mir143, Mir1930, Mir3059, Mir34b, Mir5104, Mir23b, Mir211, Mir23a, Mir672, Mir224

10	3	3	3	Mir466f-2, Mir466f-1, Mir466f-3

11	3	3	3	Mir29a, Mir29b-1, Mir29c

12	3	3	3	Mir1981, Mir340, Mir142

13	3	3	3	Mir1a-2, Mir148a, Mir148b

14	3	3	3	Mir3107, Mir486, Mir326

15	3	3	3	Mir544, Mir377, Mir1903

16	3	3	3	Mir692-3, Mir692-1, Mir692-2

17	3	3	3	Mir149, Mir3085, Mir5627

Cluster Score Nodes Edges miRNAs

**Table 2:** Cooperative miRNA modules in which miRNAs down-regulate genes.

Cluster	Score	Nodes	Edges	miRNAs
1	22.667	34	374	Mir129-1, Mir19a, Mirlet7i, Mir466l, Mir1912, Mirlet7e, Mir19b-2, Mir1961, Mir98, Mir350, Mir466n, Mirlet7d, Mir129-2, Mir1192, Mirlet7f-1, Mirlet7c-1, Mirlet7a-1, Mirlet7a-2, Mir96, Mirlet7c-2, Mir669m-2, Mir669m-1, Mir291a, Mir7-1, Mir497, Mir335, Mirlet7f-2, Mir19b-1, Mir204, Mir875, Mir883a, Mirlet7g, Mir743b, Mir743

2	14.364	34	237	Mir351, Mir5615-2, Mir93, Mirlet7b, Mir876, Mir470, Mir17, Mir677, Mir1983, Mir3063, Mir9-2, Mir320, Mir495, Mir9-3, Mir203, Mir182, Mir672, Mir449a, Mir488, Mir26a-2, Mir9-1, Mir330, Mir669k, Mir20b, Mir106a, Mir128-1, Mir466d, Mir466i, Mir218-1, Mir539, Mir883b, Mir153, Mir20a, Mir292

3	5	5	10	Mir465c-1, Mir465c-2, Mir465, Mir465b-2, Mir465b-1

4	4.75	17	38	Mir362, Mir128-2, Mir147, Mir338, Mir412, Mir125a, Mir322, Mir1941, Mir200b, Mir125b-2, Mir3097, Mir34b, Mir370, Mir125b-1, Mir133a-1, Mir133a-2, Mir1298

5	4.5	5	9	Mir124a-3, Mir5624, Mir124a-1, Mir673, Mir124a-2

6	3.333	4	5	Mir667, Mir103-2, Mir3078, Mir107

7	3.2	6	8	Mir5627, Mir3090, Mir5623, Mir346, Mir3087, Mir3113

8	3	3	3	Mir24-2, Mir149, Mir24-1

Cluster Score Nodes Edges miRNAs

## Conclusions

LPS-induced signalling must be tightly regulated to avoid excessive inflammation and to allow for tissue repair and the return to homeostasis after infection and tissue injury. Though miRNAs are critical regulators of immune responses, whether they are involved in LPS-induced signalling pathway and how is their expression regulated in mouse macrophages are still unclear. We combine mRNA expression data and miRNA-target information to infer the regulatory mechanism of miRNAs on LPS-induced signalling pathway. Also, our results suggest that miRNAs can be important regulators of LPS-induced innate immune response in BMDMs.

## Competing interests

The authors declare that they have no competing interests.

## Authors' contributions

WSW conceived the research topic and provided essential guidance. CCC performed the data collection and data processing and wrote the manuscript. All authors have read and approved the final manuscript.

## Declaractions

Publication charges of this articles was funded by National Cheng Kung University and Ministry of Science and Technology of Taiwan MOST-103-2221-E-006-174-MY2.

This article has been published as part of *BMC Genomics *Volume 16 Supplement 12, 2015: Joint 26th Genome Informatics Workshop and 14th International Conference on Bioinformatics: Genomics. The full contents of the supplement are available online at http://www.biomedcentral.com/bmcgenomics/supplements/16/S12.
